# Comparative Study of a Continuous Train of Theta-Burst Stimulation for a Duration of 20 s (cTBS 300) versus a Duration of 40 s (cTBS 600) in a Pre-Stimulation Relaxed Condition in Healthy Volunteers

**DOI:** 10.3390/brainsci11060737

**Published:** 2021-06-01

**Authors:** Jan Haeckert, John Rothwell, Ricci Hannah, Alkomiet Hasan, Wolfgang Strube

**Affiliations:** 1 Department of Psychiatry, Psychotherapy and Psychosomatics, Medical Faculty, University of Augsburg, BKH Augsburg, Dr.-Mack-Str. 1, 86156 Augsburg, Germany; Jan.Haeckert@bkh-augsburg.de (J.H.); Wolfgang.Strube@bkh-augsburg.de (W.S.); 2 Sobell Department of Motor Neuroscience and Movement Disorders, UCL Institute of Neurology, Queen Square, London WC1N 3BG, UK; j.rothwell@ion.ucl.ac.uk; 3 Department of Psychology, University of California San Diego, 9500 Gilman Drive, La Jolla, CA 92093, USA; rhannah@ucsd.edu; 4 Department of Psychiatry and Psychotherapy, University Hospital Munich, LMU Munich, Nußbaumstraße 7, 80336 München, Germany

**Keywords:** non-invasive brain stimulation, continuous theta burst stimulation (cTBS), cTBS 300 versus cTBS 600, pre-relaxed muscle condition, healthy participants

## Abstract

As variable after effects have been observed following phasic muscle contraction prior to continuous theta-burst stimulation (cTBS), we here investigated two cTBS protocols (cTBS300 and cTBS600) in 20 healthy participants employing a pre-relaxed muscle condition including visual feedback on idle peripheral surface EMG activity. Furthermore, we assessed corticospinal excitability measures also from a pre-relaxed state to better understand the potential impact of these proposed contributors to TBS. Motor-evoked potential (MEP) magnitude changes were assessed for 30 min. The linear model computed across both experimental paradigms (cTBS300 and cTBS600) revealed a main effect of TIME COURSE (*p* = 0.044). Separate exploratory analysis for cTBS300 revealed a main effect of TIME COURSE (*p* = 0.031), which did not maintain significance after Greenhouse–Geisser correction (*p* = 0.073). For cTBS600, no main effects were observed. An exploratory analysis revealed a correlation between relative SICF at 2.0 ms (*p* = 0.006) and after effects (relative mean change) of cTBS600, which did not survive correction for multiple testing. Our findings thereby do not support the hypothesis of a specific excitability modulating effect of cTBS applied to the human motor-cortex in setups with pre-relaxed muscle conditions.

## 1. Introduction

Noninvasive transcranial brain stimulation (NIBS) is a safe and effective method to investigate neuronal functioning and neuroplasticity changes in the human brain. Different stimulation protocols have been established, which are viewed to induce excitability changes of the motor cortex (M1) that outlast the stimulation interventions themselves. These effects have either been related to so called long-term potentiation (LTP)-like plasticity or long-term depression (LTD)-like plasticity [1,2]. Stimulation protocols such as transcranial magnetic stimulation (TMS), transcranial direct current stimulation (tDCS), and transcranial random noise stimulation (tRNS) typically need a conditioning of at least several minutes to induce after effects [3,4]. However, in 2005, the theta-burst stimulation (TBS) technique was introduced by Huang [5,6], and has gained attention due to its potential to induce long-lasting after effects with relatively short stimulation durations and low stimulation intensities. In TBS, a burst of 3 stimuli at 50 Hz is repeated at intervals of 200 ms. The first TBS protocols applied to humans were intermittent TBS (iTBS) and continuous TBS (cTBS) [6]. In the case of iTBS, the short 50 Hz stimulation trains are interspersed with pauses, while in cTBS, the stimulation is applied continuously. Based on animal studies reporting that short intermittent stimulation trains in the case of iTBS enhanced synaptic efficacy and led to excitability enhancing effects [7], it has been presumed that excitability changes are evoked through increased calcium influx into the postsynaptic neurons. For iTBS, it was described that the applied repeated short trains of stimulation resulted in enhanced cortical excitability in both animal studies and in humans [6,8]. The alternation between short bursts of stimulation and the pauses between them was assumed to result in the predominantly excitatory effect of iTBS. In contrast, Huang et al. described that TBS applied continuously and for a duration of 40 s (resulting in 600 pulses, hence named cTBS 600) induced excitability, diminishing after effects that lasted up to one hour following application [6]. Here, the continuous applied stimulation train was proposed to result in adaptation processes due to the increased influx of calcium, and inhibitory effects were considered to overcome excitatory effects [2,9]. In addition to these findings, continuous TBS stimulation for shorter durations of 20 s (resulting in 300 pulses, hence named cTBS 300) could be viewed as an intermediate of the iTBS and cTBS 600 paradigms, as Gentner et al. described the excitability enhancing effects of the motor-evoked potential (MEP) amplitudes lasting for about 25 min following this shorter variant of continuous TBS application [10].

Although these paradigms provide important insights for the interaction of physiological processes in the development of excitability changes, the observed after-effects following all three introduced TBS paradigms are subject to a considerable amount of variability both within and between individuals [2,11,12]. This hinders its utility as both a research and clinical tool. Various factors such as age, gender, time of day for stimulation application, attention during TBS, genetic and developmental factors as well as network activity are considered relevant contributors to TBS variability [2]. As shown elsewhere, both stimulation intensity and prior voluntary motor activation before TBS stimulation might pose controllable factors, contributing to an observed elevated intra-individual variability [10]. Of note, TBS has usually been delivered by employing a stimulation intensity equivalent to 80% active motor threshold (AMT). To determine the active motor threshold, tonic contraction of the target muscle is necessary prior to applying TBS. It has been reported that TBS applied with 80% AMT following either cTBS 300 or cTBS 600 predominantly induced excitability diminishing effects [10]. However, if participants are completely relaxed more than 10 min prior to applying cTBS 300, a mild facilitatory effect has been described, while cTBS 600 resulted in reduced excitability [10]. Furthermore, bidirectional after effects have been observed following phasic muscle contraction prior to cTBS application [13] and after administration of the L-type Ca^2+^ blocking drug nimodipine [14]. It has been proposed that after a period of rest, cTBS induces an increased Ca^2+^ influx into postsynaptic neurons via NMDA-receptors as well as L-type Ca^2+^ channels, causing excitability enhancing after effects. Furthermore, application of nimodipine prior to TBS stimulation effectively resulting in smaller amounts of Ca^2+^ influx via NMDA channels has been assumed to evoke excitability diminishing effects [2,15]. One could therefore assume that prior muscle contraction causes an activity dependent change in L-type Ca^2+^-entry, resulting in decreased MEP magnitudes. In addition to these neurophysiological mechanisms on the synaptic level, TBS after-effects have been related to contributing factors of inhibitory and facilitatory neuronal circuitry [16,17,18,19]. However, it remains unresolved as to what extent different states of the balance between inhibitory and facilitatory cortical networks contribute to TBS after-effects.

Based on this outlined current state of our knowledge, we here directly assessed the after effects of two established cTBS paradigms, cTBS 300 versus cTBS 600, in 20 healthy participants in a so called pre-relaxed muscle condition (including visual feedback of EMG activity). In contrast to previous studies, we used a pre-relaxed muscle condition to control for the potentially impeding contributory effect of muscle activation prior to cTBS. We hypothesized that in a pre-relaxed condition, after effects of both cTBS paradigms would be less variable (compared to findings in previous studies) and that we would thus obtain a clear bidirectional pattern, whereas cTBS 300 would result in significant enhancement of cortical excitability, while significant excitability diminishing after effects would be observed following cTBS 600. Furthermore, we tested a variety of cortical excitability parameters as these pose potential contributors to the expected after-effects of TBS and to better understand the physiological mechanisms underpinning the changes in excitability both following cTBS 300 and cTBS 600, respectively, from a pre-relaxed state. In this regard, we assessed short latency-intracortical inhibition (SICI), intracortical facilitation (ICF), and short-interval intracortical facilitation (SICF) as these parameters allow for assessments of cortical excitability states of the motor-cortex by means of paired-pulse TMS protocols [17]. When a subthreshold conditioning stimulus (S1) precedes a suprathreshold test stimulus (S2) at inter-stimulus intervals (ISI) of 1 to 7 ms, MEP magnitudes are diminished due to intra-cortical inhibition (SICI). Furthermore, increasing the ISI from 8 to 30 ms leads to facilitation (ICF) of MEP magnitudes [17]. In contrast, increasing the S1 intensity toward peri- and suprathreshold levels followed by an S2 stimulus at peri-threshold intensity, leads to synergistic levels of facilitation (SICF) [20]. SICF has been demonstrated to develop over short ISIs (1 to 5 ms) with three distinct peaks at ISI 1–1.5, 2.4–2.9, and >4.5 ms. Given the increasing number of clinical studies employing TBS paradigms as treatment options for psychiatric and neurological conditions such as depression [21,22,23], further insights about the sources of variability might contribute to relevant improvements in designing individualized treatment paradigms.

## 2. Materials and Methods

### 2.1. Subjects

Twenty healthy subjects (14 female, 16 right-handed, mean age: 25.3 ± 4.3) participated in this study after giving informed consent. None of the subjects had a history of neurological or mental illness or had metallic cerebral implants, nor had a history of alcohol or drug abuse and nobody was taking any neuroactive medication. The study protocol was designed in accordance with the Declaration of Helsinki and was approved by the Ethics Committee of the University College London.

### 2.2. Design

**Figure 1:**
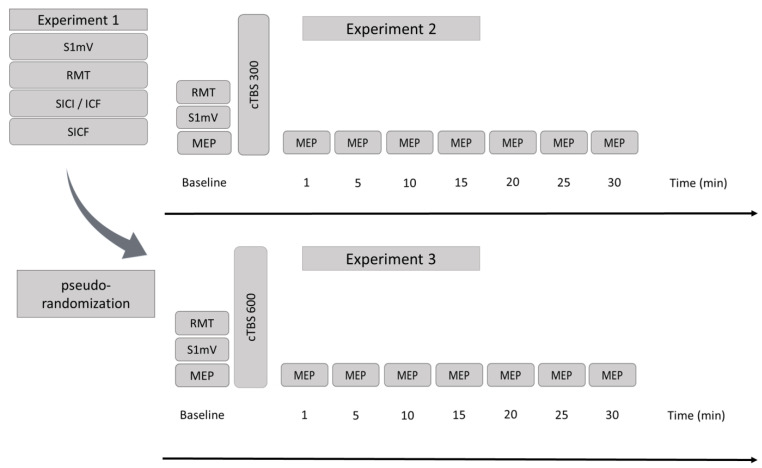
Study flowchart. Experiment 1 (**left**): S1 mV: intensity to evoke MEP of approximately 1 mV, RMT: resting motor threshold, SICI/ICF: short-intracortical inhibition/intracortical facilitation, SICF: short-interval intracortical facilitation. Experiment 2 (**upper right**): MEP: motor-evoked potentials. cTBS 300: continuous theta-burst stimulation for a duration of 20 s. Experiment 3 (**lower right**): MEP: motor-evoked potentials. cTBS 600: continuous theta-burst stimulation for a duration of 40 s. Time bins are reported in minutes. In Experiment 1, all measures (MEP, S1 mV, and RMT) were acquired using a monophasic stimulator. In Experiment 2 and Experiment 3, MEP and S1 mV were obtained using a monophasic stimulator for excitability monitoring, while RMT and a second S1 mV were acquired with a biphasic stimulator.

All 20 subjects attended three experimental sessions (all conducted by the same investigator) separated by at least three days to control for carry over effects. Before cTBS was applied, subjects took part in an additional experiment assessing various individual parameters of motor-cortical excitability (see Figure 1). After this first experimental session, participants underwent two cTBS sessions (cTBS 300 and cTBS 600) in a pseudorandomized order.

### 2.3. Experimental Procedures

During all experiments, participants were placed in a comfortable chair with their head and arms resting in a convenient position [24]. We recorded electromyographic activity (EMG) via surface electrodes on the right first dorsal interosseous muscle (FDI) of the participant’s hand and contralateral to the TMS stimulation site. Raw signals were amplified and bandpass-filtered (3 Hz–2 kHz range) using a Digitimer D-360 amplifier setup (Digitimer Ltd., Welwyn Garden City, UK) and digitized at 5 kHz using a 1401 data acquisition interface (Cambridge Electronic Design Ltd., Cambridge, UK) controlled by Signal Software (Version 5, Cambridge Electronic Design, Cambridge, UK). At the end of the study, all data were analyzed off-line using the Signal Software and NuCursor by an investigator not involved in the experiments. During the experiments, visual feedback of EMG activity was used to help participants maintain complete muscle relaxation. Assessment of motor cortical excitability was performed with a standard figure-of-eight TMS coil (70 mm, The Magstim Company Ltd., Whitland, UK) connected to a monophasic Magstim Bistim^2^ stimulator (The Magstim Company Ltd., Whitland, UK). Both cTBS protocols were delivered using the same coil design connected to a biphasic Magstim Rapid^2^ stimulator (The Magstim Company Ltd., Whitland, UK). In all experiments, the coil was held tangentially to the skull above the left primary motor cortex (M1), with the handle pointing in a dorsolateral direction at a 45° angle from the midsagittal line, leading to a posterior-anterior induced current [25]. The stimulation site that produced the largest and most stable motor evoked potentials (MEP) at moderately supra-threshold stimulation intensities was marked for each experimental session separately with a skin marker for consistent coil positioning.

### 2.4. Baseline Excitability and Monitoring of Excitability Changes

In experimental session 1, all measures were performed with a MagStim Bistim^2^ monophasic transcranial magnetic stimulator. Here, different parameters of corticospinal excitability were assessed (see Figure 1). Single-pulse TMS measurements included resting motor threshold (RMT) and the intensity to evoke MEP of approximately 1 mV peak-to-peak amplitude (S1 mV). Short-latency intracortical inhibition (SICI) and intracortical facilitation (ICF) were recorded with a standardized paired-pulse protocol (Stimulus 1: 90% RMT, Stimulus 2: S1 mV, inter-stimulus intervals (ISI): 2 ms/3 ms/9 ms/12 ms [17]). The test pulse was applied 20 times, and all paired-pulses were applied 10 times in a randomized order at 0.2 Hz. Short-interval intracortical facilitation (SICF) was evaluated with another paired-pulse protocol (Stimulus 1: S1 mV, Stimulus 2: 90% RMT, inter-stimulus intervals (ISI): 1.4 ms/1.6 ms/1.8 ms/2.0 ms/2.2 ms/2.4 ms/2.6 ms/2.8 ms/3.0 ms/3.2 ms [26]). The test pulse was applied 20 times, and all paired-pulses were applied 10 times in a randomized order at 0.2 Hz. There is good evidence that SICI relates to the activation of γ-aminobutyric acid (GABA) inhibitory circuits (GABA_A_) in the primary motor cortex [27]. The mechanisms for intracortical facilitation (ICF) are less clear, but are considered to involve the activation of excitatory cortico-cortical pyramidal cells and glutamatergic net effects [17,18,19]. The physiological origin of SICF remains to be clarified, but an intracortical origin was postulated by epidural spinal cord recordings [28]. SICF occurs at specific inter-stimulus intervals of 1.1–1.5 ms, 2.3–2.9 ms, and 4.1–4.4 ms, and if the intensity of both pulses is either around the motor threshold [20] or if a suprathreshold first pulse and a subthreshold second pulse are applied [26]. The intervals of ~1.5 ms between the facilitatory peaks closely matches the latencies between successive I-waves in epidural spinal cord recordings, therefore it was suggested that SICF reflects facilitatory I-wave interaction [20,26,29]. S1 mV for excitability measures (MEP amplitudes) before and after cTBS intervention in experiments 2 and 3 were assessed with the previously described monophasic stimulator. Single pulse MEP measurements using S1 mV stimulator intensity were obtained at baseline (40 stimuli) and after cTBS (20 stimuli each at the following time bins: 1 min/5 min/10 min/15 min/20 min/25 min/30 min) to monitor the induced after-effects (see Figure 1). The conditioned/unconditioned MEP ratio was calculated for each ISI in the case of paired-pulse measures.

### 2.5. Theta-Burst Stimulation

Continuous TBS (cTBS) was applied according to previously published protocols [6,10]. In short, each burst consisted of three stimuli with a repetition rate of 50 Hz, and the bursts were repeated with a frequency of 5 Hz. In our second experimental session, we applied a continuous train of bursts for a duration of 20 s (cTBS 300 [10]) and in the third experimental session, we applied a continuous train of bursts for a duration of 40 s (cTBS 600 [6]). The stimulation intensity for both cTBS protocols was set at 70% of resting motor threshold (RMT) elicited by a biphasic stimulator as detailed above [10]. We decided to use the RMT and not the active motor threshold (AMT) to avoid any influence of prior voluntary motor activation on after effects induced by cTBS [10,30]. Additionally, we assessed S1 mV prior to cTBS with the biphasic stimulator (see Table 1).

### 2.6. Statistical Methods

SPSS 26 for Windows (IBM, Armonk, NY, USA) was used for all analyses and level of significance was set at α = 0.05. Descriptive statistics were used to present Experiment 1 data. Two-tailed paired samples *t*-tests were computed to compare the baseline excitability measures between both cTBS experiments. To assess changes in motor-cortical excitability, an omnibus repeated measures ANOVA (RM-ANOVA) with the within-subjects factors “STIMULATION” (cTBS 300 and cTBS 600) and “TIME COURSE” (Baseline/1 min/5 min/10 min/15 min/20 min/25 min/30 min) was performed (2 × 8 design). Due to the potential variability of MEP magnitude changes following both paradigms under the outlined hypotheses, separate explorative RM-ANOVAs for cTBS 300 and cTBS 600 were performed again for the within-subjects factor “TIME COURSE” (Baseline/1 min/5 min/10 min/15 min/20 min/25 min/30 min) as well as for the average of MEP magnitude changes employing the within-subjects factor “TIME” (Baseline/average MEPs after cTBS). Sphericity was tested with the Mauchly’s test and, if necessary, Greenhouse–Geisser correction was applied. Comparisons of post-baseline time bins with the baseline were performed using LSD tests (estimated marginal means).

Next, we assessed how many participants showed expected MEP magnitude changes following either cTBS 300 (expected increase) or cTBS 600 (expected decrease), as foregoing investigations had repeatedly demonstrated variable after effects following cTBS [31,32,33,34]. For this purpose, we defined ‘expected response’ as an MEP-magnitude increase >100% relative to the individual baseline following cTBS 300 (expected facilitation [10]) and as an MEP-size decrease <100% relative to the individual baseline following cTBS 600 (expected inhibition [6]). Moreover, we tested 10% and 50% changes from the baseline, respectively, to receive more insight into the potential expected and non-expected MEP changes following cTBS.

As recent findings postulate a possible relationship between cortical excitability parameters (SICI, ICF, SICF) and intra- and inter-subject response variability following cTBS [34], we computed Pearson correlational coefficients between relative mean post MEP magnitude changes of both TBS experiments and neurophysiological factors of cortical excitability obtained in Experiment 1 (SICI, ICF, and SICF). For correlation analyses, 95% CIs were calculated according to Bonett and Wright [35].

## 3. Results

### 3.1. Baseline Characteristics

Twenty healthy subjects (14 female, 16 right-handed, mean age: 25.3 ± 4.3) participated in a total of 60 experimental sessions. To ensure comparability between both cTBS experiments, we computed paired samples *t*-tests comparing the baseline neurophysiological measures of Experiments 2 and 3. The respective analyses obtained no significant differences: biphasic RMT (*p* = 0.177), S1 mV (*p* = 0.735), baseline MEP (*p* = 0.947) (see Table 1). Descriptive analyses of parameters of baseline excitability (monophasic S1 mV, RMT, SICI/ICF, SICF, I/O) are detailed in Table 2.

### 3.2. MEP Amplitude Changes over Time

The overall 2 × 8 RM-ANOVA across both experimental paradigms (cTBS 300 and cTBS 600) revealed a main effect of TIME COURSE (F_(3.7,69.8)_ = 2.658, *p* = 0.044), but no main effect of STIMULATION (F_(1,19)_ = 0.961, *p* = 0.339), and no STIMULATION × TIME COURSE interaction (F_(4.4,84.3)_ = 0.468, *p* = 0.778). The stimulation-unspecific effect of TIME COURSE was further evaluated with LSD tests showing only an increase in MEP amplitudes at 1 min (*p* = 0.043, all other *p* ≥ 0.112).

Despite the lacking effect of STIMULATION in the aforementioned model, we added exploratory RM-ANOVAs separately for cTBS 300 and cTBS 600 based on a relevant body of literature, where both cTBS paradigms are considered to generate divergent after effects on MEP magnitude changes.

**Figure 2:**
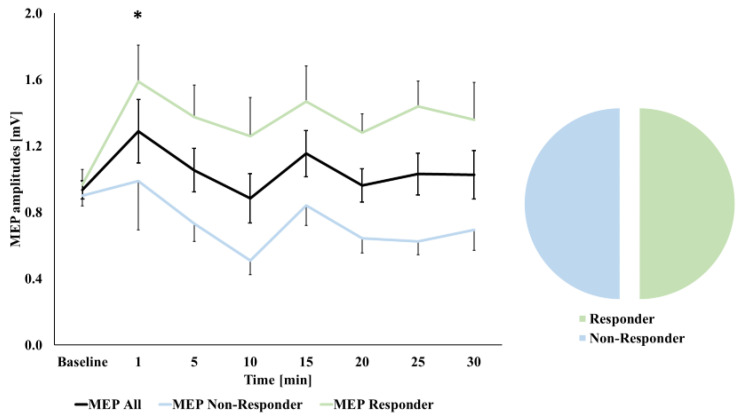
Motor-evoked potential (MEP) curves at baseline and following cTBS 300 stimulation. Time bins are reported in minutes. Black curve: MEP curve of all subjects; green curve: MEP curve of participants showing expected after effects (MEP magnitude increase; shown for a >100% threshold here); blue curve: MEP curve of participants showing unexpected after-effects. * indicates the observed significant effect (LSD test without Bonferroni correction) on the level of all participants. Error bars refer to standard error (SEM).

In the case of cTBS 300, this exploratory approach revealed a main effect of TIME COURSE (*p* = 0.031, that did not maintain significance after Greenhouse–Geisser correction (F_(3.7,70.4)_ = 2.293, *p* = 0.073). In comparison, the explorative RM-ANOVA for cTBS 600 obtained no TIME COURSE effect (F_(4.3,82.1)_ = 0.911, *p* = 0.467). To further investigate these observed differences in our exploratory analysis, we next conducted LSD tests in the case of cTBS 300, which showed higher MEP amplitudes compared to baseline only at 1 min after stimulation (*p* = 0.033, all other *p* ≥ 0.072) (see Figure 2). However, this exploratory observation did not survive correction for multiple comparisons following Bonferroni correction. As we had not obtained a significant effect of cTBS 600, the same explorative analysis was not extended to our second stimulation paradigm.

As a next step, we computed the average of MEP magnitude changes across all time bins and compared this measure to MEP magnitudes at baseline. For both stimulation paradigms, the two respective exploratory RM-ANOVAs comparing the baseline to the mean average post-stimulation MEPs obtained no significant main effects of TIME (cTBS 300: F_(1,19)_ = 1.861, *p* = 0.188; cTBS 600: F_(1,19)_ = 0.084, *p* = 0.775).

**Figure 3:**
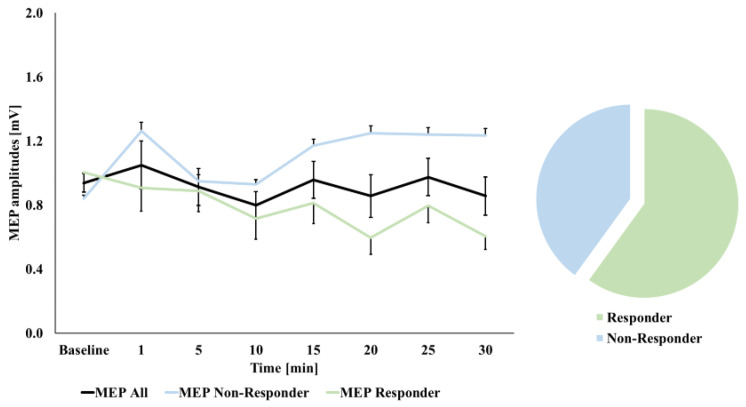
Motor-evoked potential (MEP) curves at baseline and following cTBS 600 stimulation. Time bins are reported in minutes. Black curve: MEP curve of all subjects; green curve: MEP curve of participants showing expected after effects (MEP magnitude decrease; shown for a <100% threshold here); blue curve: MEP curve of participants showing unexpected after-effects. Error bars refer to standard error (SEM).

Finally, with respect to the above-described response analysis (see statistics section), we observed the expected response in 10 participants (50%) following cTBS 300 (see Figure 2). In the case of cTBS 600, we found an expected decrease in MEP magnitudes in 12 participants (60%, see Figure 3). Different approaches to define response to cTBS by higher/lesser thresholds (namely ≥ 110% and ≤90%, and ≥150% and ≤50%, respectively) resulted in gradually decreasing observations of expected response: in the case of a threshold of ≥110% and ≤90%, respectively, we again observed the expected response in 10 participants (50%) following cTBS 300; however, in the case of cTBS 600, the expected decrease in MEP magnitudes was only observed in seven participants (35%). A more rigorous threshold of ≥150% and ≤50%, respectively, resulted in only four participants (20%), showing an expected increase in MEP magnitudes following cTBS 300 and only one expected responder (5%) in the case of cTBS 600.

### 3.3. Correlations with Baseline Excitability Measures

**Figure 4:**
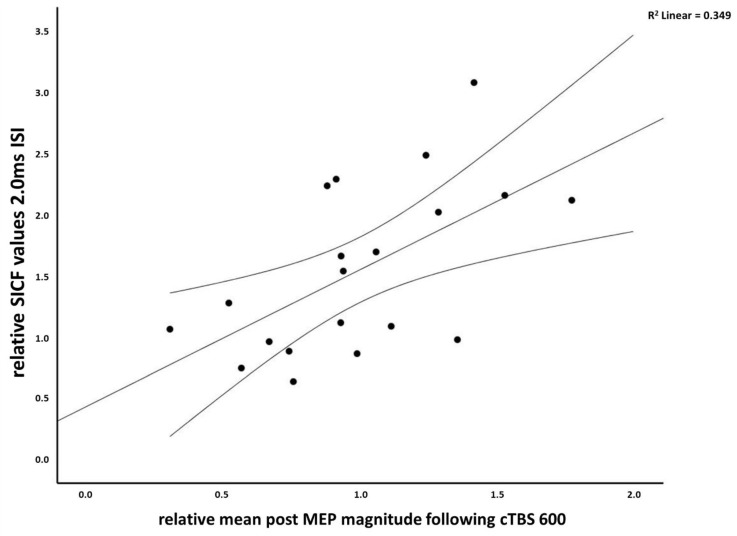
Pearson correlational coefficient between relative mean post MEP magnitudes following cTBS 600 and relative SICF values at ISI 2.0 ms (r = 0.591, *p* = 0.006) including mean 95% confidence intervals.

For cTBS 300, the Pearson correlational coefficients obtained no significant correlations between relative mean post MEP and SICI (all |r| ≤ 0.269, all *p* ≥ 0.251), ICF (all |r| ≤ 0.072, all *p* ≥ 0.762), or SICF at any ISI (all |r| ≤ 0.202, all *p* ≥ 0.392). In contrast, for cTBS 600, positive correlations were obtained between relative mean post MEPs and SICF in the case of ISI 1.4 ms (r = 0.466, *p* = 0.038, 95% CI [0.030, 0.753]), and ISI 2.0 ms (r = 0.591, *p* = 0.006, 95% CI [0.201, 0.819]) (see Figure 4) (all other ISI: all |r| ≤ 0.398, all *p* ≥ 0.082), while no significant correlations were observed for SICI (all |r| ≤ 0.404, all *p* ≥ 0.077) and ICF (all |r| ≤ 0.132, all *p* ≥ 0.580). However, none of the observed significant correlations survived corrections for multiple comparisons following Bonferroni correction (adjusted *p*-value 0.003, see Table 2 for descriptive statistics of the analyzed variables).

## 4. Discussion

With this study, we present experiments comparing the after effects induced by cTBS 300 and cTBS 600, respectively, in a cohort of 20 participants using an intensity of 70% RMT and a pre-relaxed muscle condition. Overall, our main analyses did not show a specific stimulation effect of two different cTBS protocols on motor-cortical excitability. Analyses showed a significant effect on the change of MEP amplitudes over time, but subsequent analyses only confirmed a subtle stimulation-independent increase in MEP amplitudes compared to baseline immediately after stimulation. Thus, our overall finding must be considered as a negative finding.

In contrast to our findings, Gentner et al. showed continuously increased MEP magnitudes at 16 min and 24 min after cTBS 300 stimulation using a stimulation intensity of 70% RMT in 16 participants [10]. Similarly, Doeltgen et al. showed a significant increase in MEP magnitude at 30 min following cTBS 300 in their study on 16 subjects using a stimulation intensity of 70% RMT [36]. Interestingly, authors were not able to show an effect when stimulation intensity was adapted to 65% RMT [36]. Contrary to these findings, Stefan et al. described a significant MEP-decrease directly after the stimulation in seven subjects following cTBS 300 with an intensity of 70% RMT [37]. Furthermore, Fang et al. used an intensity of 80% AMT in nine participants and showed a significant MEP-decrease following cTBS 300 [38]. A consecutive meta-analysis summarized that stimulation with cTBS 300 induced mainly inhibitory after effects as it was also first described by Huang [1,6]. However, in this meta-analysis, most of the cTBS 300 protocols were performed with a stimulation intensity set to the AMT and not with RMT as in our experiments. These differences of muscle pre-activation prior to cTBS might have significantly contributed to the inter-study differences [10,13,39].

Furthermore, in the case of cTBS 600, while long-term depression of the MEP amplitude would have been expected [1,2,6,10], we were not able to obtain significant after effects. In contrast, Huang et al. reported reduced excitability after effects that lasted up to one hour following application [6]. Furthermore, Stefan et al. were able to show MEP magnitude decreases at 5 min and at 35 min following cTBS 600 with a stimulation intensity of 70% RMT in a cohort of 18 subjects [37]. Moreover, Di Lazzaro et al. also reported a significant decrease in MEP amplitudes directly after stimulation using an intensity of 80% RMT. However, this effect was abolished 30 min after the stimulation [40]. In addition, Doeltgen et al. tested cTBS 600 with 80% AMT in 14 participants and reported significant MEP magnitude decreases at 20 min and 30 min following stimulation [41]. Finally, Goldsworthy et al. observed significant reductions in MEP amplitudes directly after stimulation (0 min and 10 min) in 16 subjects when using cTBS 600 and a stimulation intensity of 70% RMT. Again, these effects had normalized at the second measurement taken between 30 and 40 min following stimulation [42].

Of note, while some previous experiments reported different after effects, our findings of no excitability-diminishing effect of cTBS 600 were in line with a number of previous experiments. In a study on 56 participants employing cTBS 600 stimulation with an intensity of 80% AMT, Hamada et al. were also not able to show a significant after effect [43]. In this cohort, a high inter-individual variability of the induced after effects was observed and the authors were capable of predicting about 50% of the expected inhibition after cTBS stimulation, when subjects showed a larger MEP latency difference evoked by different current directions, which is thought to reflect the recruitment of different neuronal populations [43]. Additionally, Hordacre et al. did not report a significant after effect of cTBS 600 at an intensity of 70% RMT in a cohort of 34 subjects [44]. At the same time, they observed a correlation of baseline MEP variability and cTBS 600 response, where subjects with higher baseline MEP variability showed a stronger inhibitory response [44]. In a second experiment, the authors further investigated I-wave recruitment and MEP variability and reported a significant correlation between AP-LM latency difference and cTBS response, which did not survive correction for multiple comparison [44].

In our corresponding analyses evaluating the relationship between SICF and the after-effects, we were able to show an exploratory correlation between SICF at 1.4 ms and 2.0 ms and MEP-changes following cTBS 600, which did not survive Bonferroni correction. Further, for the 1.4 ms ISI the lower end of the confidence interval is 0.03, which indicates that this correlation may not be present in the population. The 1.4 ms correlation must thus be interpreted with caution. While exploratory, this analysis indicated for the first time that participants showing less excitation with the SICF protocol might also display the expected excitability diminishing after effects followed by cTBS 600 stimulation. This new finding is in line with previous reports also showing a potential association between I-wave recruitment and cTBS after effects.

Considering the findings that subjects in whom late I-wave circuits were likely activated by TMS were more likely to respond in the expected direction with TBS [43] and the association between cTBS response and late I-wave recruitment [44], our observation of a potential correlation between reduced early SICF responses and excitability diminishing effects of cTBS 600 (see Figure 4) might further contribute to identifying another factor impacting cTBS variability. One could conclude that I-wave recruitment might pose a measurable variable that might help to determine and facilitate expected response to inhibitory motor-cortical cTBS paradigms.

In an overview of our observations, our study bears the limitation that the overall after-effects of both cTBS stimulation paradigms were not consistent with our outlined hypotheses. This could—at least in part—be explained by the so far reported high rates of inter-individual variability following both employed TBS paradigms or our sample size of 20 participants. In context with recent respective considerations, an even larger sample size of ≥30 participants might help to show more reliable subgroup differences and overall group level effects [2] and future research efforts using cTBS should include these considerations. Additionally limiting is the fact that baseline characteristics were measured not at the same day of TBS application. Finally, our main analyses provided a negative result—all subsequent analyses must be considered as exploratory that would not survive corrections for multiple testing. Thus, these findings must be confirmed in independent samples.

However, a specific advantage of our study is that, so far, it composes the largest experimental study comparing the after-effects induced by both cTBS 300 and cTBS 600, respectively, in a cohort of healthy subjects and a pre-relaxed muscle condition, and that we were able to relate our findings in an exploratory analysis to new potential baseline predictors of cortical excitability. In this regard, we obtained a relationship between early SICF responses and expected after effects of cTBS 600. As neurophysiological findings such as these might pose a potential means of identifying people that respond to cTBS in the desired way, further research efforts appear relevant here, especially for clinical applications.

## Figures and Tables

**Table 1:** Baseline motor-evoked potentials (MEP, monophasic), resting motor thresholds (RMT, monophasic in Experiment 1 and biphasic in Experiments 2 and 3), and intensities to evoke MEP of approximately 1 mV (S1 mV, monophasic in experiment 1 and biphasic in Experiments 2 and 3). To ensure comparability between both cTBS experiments, we computed paired samples *t*-tests comparing the baseline neurophysiological measures of Experiments 2 and 3. The respective analyses obtained no significant differences. Note: S1 mV data of one participant were excluded due to incomplete data acquisition. Data are shown as mean values ± standard deviation. mV: millivolt; %: percentage of stimulator output.

	Experiment 1	Experiment 2 cTBS 300	Experiment 3 cTBS 600	Dependent Samples *t*-Tests (Experiment 2 vs. Experiment 3)	
				Test Statistic	*p*-Values
Baseline-MEP [mV]	-	0.934 ± 0.244	0.938 ± 0.262	t_(19)_ = 0.067	0.947
RMT [%]	47.25 ± 8.30	50.30 ± 9.69	49.05 ± 9.90	t_(19)_ = 1.403	0.177
S1 mV [%]	55.85 ± 10.42	58.00 ± 10.58	57.68 ± 9.29	t_(18)_ = 0.344	0.735

**Table 2:** Descriptive statistics of baseline excitability measures (Experiment 1: S1 mV, RMT, SICI, ICF, SICF) and for relative mean post stimulation MEP magnitudes of Experiment 2 (cTBS 300) and Experiment 3 (cTBS 600). Relative values (paired-pulse/test pulse) are reported for short-intracortical inhibition (SICI), intracortical facilitation (ICF) and short-interval intracortical facilitation (SICF). All measures were obtained using 90% RMT stimulation intensity for the conditioning stimulus in paired-pulse protocols. ISI: inter-stimulus interval, RMT: resting motor threshold (monophasic), S1 mV: intensity to evoke MEP of approximately 1 mV (monophasic). Data are shown as mean values ± standard deviation.

	ISI	N	Mean ± SD
S1 mV		20	55.85 ± 10.42
RMT		20	47.25 ± 8.30
SICI	2 ms	20	0.75 ± 0.45
	3 ms	20	0.89 ± 0.70
ICF	9 ms	20	2.16 ± 1.28
	12 ms	20	2.22 ± 1.17
SICF	1.4 ms	20	3.44 ± 1.47
	1.6 ms	20	3.43 ± 2.10
	1.8 ms	20	2.09 ± 1.33
	2.0 ms	20	1.54 ± 0.69
	2.2 ms	20	1.36 ± 0.55
	2.4 ms	20	1.97 ± 0.66
	2.6 ms	20	2.57 ± 0.76
	2.8 ms	20	2.13 ± 0.69
	3.0 ms	20	2.17 ± 0.99
	3.2 ms	20	1.87 ± 1.01
cTBS 300	mean post rel. MEP	20	1.12 ± 0.42
cTBS 600	mean post rel. MEP	20	0.99 ± 0.36

## Data Availability

Data are available upon reasonable scientific request.
